# Abortion debates in Finland and the Republic of Ireland: textual analysis of experiential thinking and argumentation in parliamentary and layperson discussions

**DOI:** 10.1186/s12978-017-0418-y

**Published:** 2017-12-02

**Authors:** Anne-Mari Mustonen, Tommi Paakkonen, Esko Ryökäs, Petteri Nieminen

**Affiliations:** 1 University of Eastern Finland, Faculty of Health Sciences, School of Medicine, Institute of Biomedicine/Anatomy, P.O. Box 1627, FI-70211 Kuopio, Finland; 2 University of Eastern Finland, Faculty of Science and Forestry, Department of Environmental and Biological Sciences, P.O. Box 111, FI-80101 Joensuu, Finland; 3 University of Eastern Finland, Philosophical Faculty, School of Theology, P.O. Box 111, FI-80101 Joensuu, Finland

**Keywords:** Argumentation, Confirmation bias, Experiential thinking, Fallacy, Induced abortion, Narrative, Political rhetoric, Testimonial

## Abstract

**Background:**

The ethical discussion about abortion has been polarized in Finland and the Republic of Ireland, two European countries with very different abortion legislation (liberal vs. highly restrictive). The aim of the present study was to analyze experiential thinking patterns and argumentative strategies in political and layperson debates regarding induced abortion.

**Methods:**

The content of Finnish and Irish texts (*n* = 493), consisting of transcripts of parliamentary debates and online texts, such as blogs, was analyzed systematically. The texts were investigated for the aspects of experiential thinking, for selected argumentative moves and for any differences in the prevalence of these features between countries or between political vs. layperson debates.

**Results:**

The Finnish and Irish discussions about induced abortion relied heavily on experiential thinking patterns and emotionally laden arguments instead of objective research data. This was evident in the very high prevalence of testimonials, narratives, loaded language and appeals to emotion in both political and layperson debates regardless of the country or the debater's position on abortion issue. Research data that did not support the position of the debater were relatively often omitted by confirmation bias. The Irish debaters appealed to popularity more often than the Finnish ones, while magical/religious thinking was mainly observed in the Finnish layperson discussion. The national history and the prevailing cultural and religious atmosphere of the two countries could explain these differences.

**Conclusions:**

The abortion debate mostly reinforces the opinions of one's peer group rather than convinces the opposite party to change their position. The stalemate and continuation of the same arguments being repeated could be associated with experiential thinking and emotional argumentative strategies in both political and layperson debates.

## Plain English summary

The ethical discussion about abortion is polarized in Finland and the Republic of Ireland, two European countries with liberal and highly restrictive abortion legislation, respectively. The aim of the present study was to analyze abortion debates from these countries. The content of parliamentary transcripts and online texts, such as blogs, was analyzed systematically. The texts were investigated for intuitive thinking patterns, emotional argumentative strategies and for any differences in the occurrence of these features between countries or between political and layperson debates. The studied debates relied heavily on experiential thinking and emotionally laden arguments instead of objective research data. This was evident in the very high prevalence of testimonials, narratives, loaded language and appeals to emotion in both political and layperson debates regardless of the country or the debater's position on abortion issue. Research data that did not support the position of the debater were relatively often omitted by confirmation bias. The Irish debaters appealed to popularity more often than the Finnish ones, while magical/religious thinking was mainly observed in the Finnish layperson discussion. The national history and the prevailing cultural and religious atmosphere of the two countries could explain these differences. The abortion debate mostly reinforces the opinions of one's peer group rather than convinces the opposite party to change their position. The results suggest that the stalemate and continuation of the same arguments being repeated could be associated with experiential thinking and emotional argumentative strategies in both political and layperson debates.

## Background

The ethical discussion about abortion focuses on two complex issues: *i*) if the embryo/fetus is from fertilization or at some stages of pregnancy unequivocally entitled to protection of life and *ii*) if the pregnant woman is obliged to allow the embryo/fetus to use her body on some or all occasions [1]. Because an indisputable ethical conclusion has not been reached, the freedom of conscience in many liberal societies includes regulated abortion. In contrast, anti-abortion advocates regard the fetus as a person from the moment of conception and consider the termination of pregnancy a form of homicide. Principal Christian denominations as well as other major religions oppose induced abortion [2]. They mostly reserve the right to life-threatening situations of the pregnant woman but are more diverse when it comes to other indications.

The aim of the present study was not to discuss whether it can be morally right or wrong to terminate a pregnancy but to perform a textual analysis on opinions for and against abortion or conscientious objection (CO; refusal by medical professionals to participate in abortion procedures due to religious or ethical reasons). The analysis was conducted for two European countries, Finland and the Republic of Ireland (hereafter, Ireland), that both have quite similar population sizes and health care systems [3, 4] but very different abortion legislation [5, 6]. Ireland is considered a morally conservative country [7] with over 78% of the population identifying as Catholic [8]. Finland can be considered more liberal [9], even though 72% of the population belongs to the national Evangelical Lutheran Church [10]. Obviously, this characterization oversimplifies the situation of the nations and, instead of being uniform, both countries do have voices that are in opposition to the general national ethos.

Abortion remains criminally prohibited in Ireland except in cases where a pregnant woman's life is at risk, including suicidality [6, 11]. Abortion is not legal in cases of rape, incest or life-limiting conditions of the fetus. A person found guilty of intentionally destroying unborn human life is liable to a possible maximum sanction of 14 years imprisonment, but we were unable to locate any data indicating prosecutions. Every year, thousands of Irish women seek abortion abroad, mainly in the UK, and many others purchase abortifacients from Internet sources [7, 12]. In contrast, the Finnish health care system grants abortion virtually on demand until the 12th gestational week, but the pregnant woman is obliged to provide justification, why the continuation of pregnancy would be a significant burden (so called “social indications”) [5, 13, 14]. While at first glance this is different from the more permissive laws of many countries in Western Europe (e.g., Sweden; [15]), in reality the difference is minor, as practically any justification from the woman is accepted during this period. After the 12th week, the criteria mostly include medical conditions of the woman or the fetus, and social indications are no longer allowed except by the permission of the National Supervisory Authority for Welfare and Health. Between weeks 20–24, only serious medical conditions of the fetus are mentioned as indications. In Finland, 96% of the abortions are performed medically [16]. Unlike in many other European countries, such as Ireland, there is no CO to participating in abortion procedures in Finland [6, 13, 17].

To assess the polarized debate on abortion, it would be interesting to examine, how the opposite views are presented in public discourse and how they reflect the thinking patterns of the participants. Generally, there are two interactive information-processing systems: rational and experiential [18, 19]. The latter is considered evolutionarily old and rapid in everyday situations, where it is crucial to organize and interpret information automatically. Experiential thinking is intuitive and emotional, and its use can cause errors of judgment. This approach prefers concrete information, often in the form of *i*) personal or anecdotal experience (testimonials and narratives) [18–20]. Due to *ii*) confirmation bias, a person seeks information consistent with previously existing beliefs while alternative hypotheses are not readily considered. In addition, experiential reasoning is characterized by *iii*) generalization and stereotypical thinking as well as by *iv*) magical beliefs. A morally neutral issue is often given *v*) moral significance. Beliefs based on experiential thinking are *vi*) resistant to change, and logical evidence and contradictory information have only little influence on them.

Both experiential thinking and fallacies have been suggested to create and enforce false beliefs [19, 21]. Fallacies are violations of rules for critical discussion, which aims to resolve a difference of opinion [22]. The evaluation of arguments by an audience can be affected by fallacies, especially in a case of pre-existing biases [23]. Both experiential thinking and fallacies have a tendency towards generalizations and simplification of complex data [18–20]. In addition, the use of personal experience or narratives, which often take the form of testimonials or appeals to authority, is shared by both, as is the habit of giving moral significance to neutral issues. These similarities provide fertile ground for assessing texts on morally ambiguous issues that are strongly opposed to each other. Regarding the abortion discourse, it is possible that both sides fail to concentrate on available unbiased research data but utilize emotional arguments and experiential thinking. This could cause failure in communication and a stalemate evidenced by the continuous reappearance of the same arguments. It would be of benefit to break this vicious circle of conflict.

The use of experiential and scientific/medical arguments has also been examined previously in abortion debates but with different suppositions [24, 25]. Data originating from medical sciences have been observed to form a basis for emotional argumentation and persuasion [26–29]. The emotional appeal of ultrasound images, turning of fetal images into a narrative, and appeals to authorities may contain material that originate from science but the actual discourse is not scientific any longer. The present study has a novel approach by making a clear distinction between objective research data and emotional argumentation. This does not mean that emotional arguments would be invalid or that the experiences of people providing the testimonials would not be sincere, but they represent a different type of material, which cannot necessarily be validated from independent sources or by repeating an experiment. Similarly, research data are not always interpreted objectively, but the validity of these arguments can be tested in case of uncertainty, as data based on the scientific method can be returned to or reproduced with the methodology described; and even the validity of the conclusions can be reassessed. Basically, it is always possible to go to the original publication and data to assess if research results were obtained according to the norms of science. In comparison to anecdotal evidence or testimonials, research findings provide a data bank that can be accessed and evaluated. This allows others to do fact checking independent of the people performing the original study—although this is obviously not always conducted in a perfect or objective manner.

Little previous research has been conducted on abortion discourse by politicians vs. laypersons, and the aim of the present study was to identify typical argumentative moves and patterns of experiential thinking in political debate and layperson discussion regarding induced abortion. Two European countries with relatively similar population sizes, governance and health care systems were chosen to see how the cultural and religious differences in Finland and Ireland would influence abortion discussions. If political decision-making were based on non-rational argumentation, it could be difficult to establish legislation applicable not only to one’s peer group but also to those who do not share the same ethical or moral background. As political decision-making should advance the well-being of all citizens, it could be expected of politicians to be able to assess also data that do not support one’s own worldview or preconceptions. It was hypothesized that *i*) the occurrence of emotional argumentation and features of experiential thinking would be high in abortion discourse as the subject raises strong emotions in people and that *ii*) parliamentary discussion would contain fewer cases of emotional argumentation and features of experiential thinking than layperson debate.

## Methods

The content of 493 online texts and official transcripts of parliamentary debates on abortion was analyzed systematically. The sampled texts represented *i*) discussions at the Parliament of Finland (*n* = 166, focus on the years 2013–2015, range 2006–2015) and *ii*) the lower house of Oireachtas (Dáil Éireann) of Ireland (*n* = 122, focus 2013–2015, range 2013–2015) and *iii*) online texts in Finnish (*n* = 101, focus 2010–2015, range 2005–2016) and *iv*) in English (*n* = 104, focus 2013–2015, range 2008–2016) from the same countries. These texts were not randomly selected but represented available parliamentary transcripts and online texts obtained by search engines using abortion-related keywords. The transcripts of parliamentary debates were browsed on https://www.eduskunta.fi/FI/search/Sivut/vaskiresults.aspx and http://oireachtasdebates.oireachtas.ie/debates%20authoring/debateswebpack.nsf/fulltextsearch?readforms by using the keywords “abortion” and “termination” (“abortti”, “raskaudenkeskeytys” in Finnish) on the websites’ search engines. While the general framework of discussion was the same (abortion), the specific laws being debated differed (Finland: main focus on allowing CO for medical professionals; Ireland: allowing abortion in specific circumstances). Moreover, the Finnish transcripts tended to be shorter than the Irish ones, caused at least partly by more strict time limits. Regarding layperson debates, several types of online texts, such as blogs*,* were retrieved by using selected keywords in Finnish and English (“abortion”, “abortion blog”, etc.) with Internet search engines and by browsing platforms representing Finnish and Irish writers. This yielded material from the home pages of private persons and politicians, subscription newspapers, Internet news publications and pro-life/pro-choice organizations. A minority of texts was anonymous. All the selected documents were public, and the authors were not contacted to gain permission for the analysis. The texts were not classified into pro-life and pro-choice, as many authors revealed a position in between these categories.

The analysis of experiential thinking was based on previous literature on the subject [18–20]. The selected features were classified in this study as follows: personal or anecdotal experience (testimonials, narratives and metaphors) as the principal tool to assess data, confirmation bias (seeking information consistent with existing beliefs), stereotyping/generalization (constructing a standardized mental picture of a group that represents an oversimplified opinion/simplifying complex information) and magical/religious beliefs (referring to supernatural phenomena as relevant for the argument, including religious argumentation for secular legislation). Argumentative strategies were spotted in the discursive moves and classified by using several, partly divergent sources for argumentation and fallacies [22, 23, 30–36]. The context and validity of the arguments were assessed and, eventually, an argument was regarded fallacious, if it highlighted aspects that were irrelevant to prove or disprove a claim, for instance, characteristics of a person instead of content that would be pertinent for a writer’s position. The nature of the debate was taken into consideration, as argumentative moves that would be fallacious in some other contexts can be considered sound in political debate [30, 32]. For instance, *ad consequentiam* was regarded as fallacious in cases the alleged consequences of a bill were unsupported by or lacking any evidence, but it was accepted that these examples could be equally well assessed to represent reasonable argumentative strategies in political decision-making [30, 31]. Detailed descriptions of the fallacies chosen for the analysis are available in the referenced literature [22, 23, 30–36]. Regarding experiential thinking, the feature of attaching moral labels to neutral issues was not included in the analysis as abortion debate was accepted to be a discussion about ethics. In several cases, the classification of arguments and that of experiential thinking overlapped, i.e., the same passage could be assessed to be both fallacious and to contain features of experiential thinking.

### Statistical analyses

The prevalence of features of experiential thinking and selected argumentative strategies were calculated by documenting their occurrence in the sampled texts. Multiple occurrences within a text were not recorded due the large variation in text lengths. The distribution of prevalence was analyzed with the χ^2^ test or, if the test criteria were not met, with the Fisher’s exact test using the IBM SPSS *v*21.0 program (IBM, Armonk, NY, USA). The *p* value <0.05 was considered statistically significant. The results are presented as the percentage of texts within a category that contained at least one occurrence of a feature of experiential thinking or an argumentative move.

Statistical comparisons were also performed between the political parties the deputies of which held the most numerous speeches: Christian Democrats (political position: center to center–right, *n* = 54), Finns Party (social: right-wing, economic: center–left, *n* = 39), Social Democratic Party of Finland (center–left, *n* = 21), National Coalition Party (center–right, *n* = 15) and Left Alliance (left-wing, *n* = 14) for Finland and Fine Gael (center–right, *n* = 45), Fianna Fáil (center–right, *n* = 12), United Left (left-wing, *n* = 10) and Labour Party (center–left, *n* = 9) for Ireland, which also had one study group of independent politicians (*n* = 23). The results were also compared between all center–left-wing and center–right-wing parties, separately for both countries, to see whether political spectrum affected the occurrences of features of experiential thinking or argumentative strategies.

## Results

### General results

**Fig. 1:**
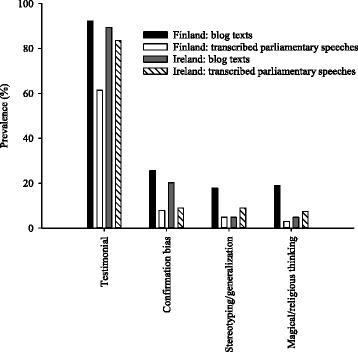
Prevalence of features of experiential thinking in the sampled material (*n* = 90–166 texts/group). For the “Finland: blog texts” group, the blogs written by politicians were excluded and only layperson authors were included

Features of experiential thinking were abundant in the study material (Fig. 1; Table 1). Testimonials were documented in 79% of all sampled texts. They were either personal experiences, narratives or assertions without any further justification, as well as testimonials of others cited as a part of the debater’s arguments. Metaphors were also included in this category. Confirmation bias (14%), stereotyping/generalization (8%) and magical/religious thinking (7%) were less popular.

**Table 1:** Examples of features of experiential thinking in the studied abortion debate

Experiential thinking	Examples^a^	Reference
Personal testimonial	Abortion solves nothing. I know a number of women who have had abortions and deeply regretted it. I genuinely do not know any woman who has had a baby and regretted it.	[57]
Testimonial of others	Most of the women and health professionals Amnesty spoke to, said that a woman’s rights inevitably come second. Lupe, a woman who was forced to carry a dead foetus for 2 months, told us: “When a woman gets pregnant in Ireland, she loses her human rights.”	[58]
Metaphor	Amnesty [International] swallowed a camel – but strained at a gnat!	[59]
Confirmation bias	Three years ago, on the first occasion on which I introduced legislation to deal with some of the issues involved, I received a letter from a Church of Ireland bishop in Tipperary in which he congratulated me on taking a stand and indicated that he was sick of the systemic spinelessness of the political establishment. What he wrote came back to me as I listened to the contributions to this debate and it sums up where we stand. Not a single credible argument against the Bill has been put forward.	[60]
	We know that post-abortive stress syndrome is a significant threat to women’s mental health.	[61]
	I, for my part, am disappointed with this report of the committee majority and I do marvel at these justifications, because we know that in almost all other countries in western Europe, except Sweden and Finland, this legislation [conscientious objection regarding the abortion procedure] works. It simply works.	[62]
Stereotyping/Generalization	I am struck by a terrible irony that Mrs. Halappanavar and her husband probably came to Ireland for a better life than they would have had in India. Moreover, they came to a country with very fine medical facilities and in which pregnant women are very well looked after. They came from a country where women are not treated in an equitable way, in which there are forced child brides and in which the caste system leads to girls and women being treated appallingly. Consequently, it is very sad that Mrs. Halappanavar lost her life here, as is the reason.	[63]
	But the greatest blame lies in these social abortions. There is no medical or any other reason, something just went askew, and then after one mistake, you make an even bigger one.	[64]
	Abortion is not a medical treatment; it is a social reaction to a culture which says that says sexual freedom is all and must be protected to the utmost, up to and including abortion.	[65]
Magical/Religious thinking	Human life is sacred, it must be protected under all circumstances.	[66]
	Children are a gift from God. You should treat them accordingly.	[67]
	Because God created man and set the womb of the woman as the place for the birth of life, it is clear that if a tiny human being is killed through abortion, it will have a harmful effect on the woman who committed abortion as well as on her body.	[68]

^a^the Finnish examples were translated by the authors

**Fig. 2:**
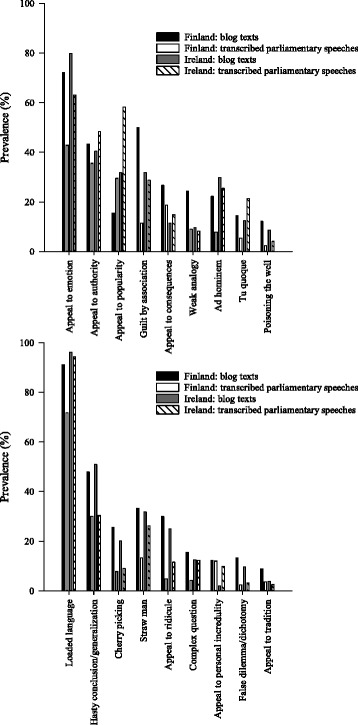
Prevalence of selected types of arguments in the sampled material (*n* = 90–166 texts/group). For the “Finland: blog texts” group, the blogs written by politicians were excluded and only layperson authors were included

The most common types of arguments in the studied abortion debates can be seen in Fig. 2. They included in the descending order of prevalence: loaded language (87% of texts), appeal to emotion (62%), appeal to authority (41%), hasty conclusion/generalization (38%), appeal to popularity (35%), guilt by association (28%), straw man (25%), ad hominem (20%), appeal to consequences (18%) and appeal to ridicule (15%). Some examples of these types of arguments are presented in Table 2.

**Table 2:** Examples of potential fallacies in the studied abortion debate

Fallacy	Example^a^	Reference
Appeal to authority	Based on the Word of God (*Bible*) and sound ethics it is clear that abortion is not right.	[68]
	I am not going to read here, what Mother Teresa herself said during the acceptance speech of the Nobel Prize on December 11, 1979, but I am going to say that what she said influenced my conviction on this issue 35 years ago, and all that is still valid.	[69]
Ad hominem	The Minister is a young man and he should wise up.	[70]
	My last word is directed at Fianna Fáil, which has recently decided that, apparently, the eighth amendment does not matter. It would seem its members have decided to position themselves as backwoodsmen. No wonder they do not represent women. They clearly are out of touch with the general population. They should change their position and grow a spine on behalf of women in this country.	[71]
Appeal to ridicule	The only response the Minister gave on this issue when we discussed it previously was that if we were to remove the eighth amendment, we would remove all protections for women. That is bizarre. It is as if suddenly women were going to be the victims of some rampaging murderers or whatever.	[72]
	It must be a strange place, that parallel universe of the pro-abortion campaigner, where killing is dressed up as compassion, and where, in stout denial of all the scientific facts, babies aren’t really human beings. Pregnant women aren’t really carrying a baby in their wombs you see. Maybe they find them in cabbage patches. Or storks bring them in cute colourful slings.	[73]
Guilt by association	Deputy Wallace referred to the possibility that 30% of people in prison are wrongly convicted. I do not know if that is true. I certainly hope it is not, but a miscarriage of justice can be reversed, and people can be released. Terminations are not reversible. One of the reasons we do not have the death penalty in this country is exactly for that reason.	[74]
	I have listened to some of the contributions from some of the people who would have opposing views to mine, talking about a free vote and conscience, etc. I ask these people, who talk about wrestling with their consciences on this Bill which will protect women in difficult circumstances, where their conscience was during treatment of the women and girls in the Magdalen laundries. Where was their voice during the clerical sexual abuse which went on for 80 or 90 years? They were not to be heard.	[75]
Appeal to consequences	The fetus is a Homo Sapiens with human dignity. If this is denied, the universal and objective value of human dignity is also denied. In that case, there’s ethically an open road towards, for example, involuntary euthanasia, eugenics and mass murders.	[76]
	If we go down that road, where a person can decline from performing a duty that (s)he has accepted, for ethical reasons, we’ll very soon be in the situation where, for instance, a taxi-driver can decline from taking a woman to a hospital, if it is possible that she’s going there for an abortion.	[77]
Weak analogy	Based on conscience, we allow people to decline from taking the military service, although they are just learning there how to kill.	[78]
	Amnesty and others would claim that abortion is a matter of “choice”. We do not give people a choice when it comes to issues like smoking in public places, drinking-driving, wearing seat-belts, stealing, slander and libel, selling hardcore drugs, raping or killing. We do not agree to “choices” in such circumstances because such things are a danger to safety, health and human dignity. I would argue that the same may be said with regard to abortion.	[79]
Straw man	Deputy O’Riordain, it seems, wants our morality to revert all the way back to the Roman Empire.	[80]
	Now this initiative seems to be used to gain something totally different: by using the health care personnel, people are trying to create for our society a climate that would make abortion a shame and a taboo.	[81]
Complex question	Why is only the life of an unborn child holy to the men of Finns Party?	[82]
	How much longer can the political establishment in this country hold to a barbaric medieval law which equates a woman with a foetus and leads to these situations?	[71]

^a^the Finnish examples were translated by the authors

### Comparisons between laypersons and politicians

Regarding the features of experiential thinking, the prevalence of confirmation bias was higher for layperson than political opinions in both countries (Fig. 1). The same was observed for testimonials (including narratives and metaphors), stereotyping/generalization and magical/religious thinking in Finland.

There were several differences in the prevalence of argumentative moves between the layperson and political debates (Fig. 2). The prevalence were higher in layperson opinions for appeal to emotion, hasty conclusion/generalization, cherry picking and appeal to ridicule in both countries. In contrast, politicians utilized appeal to popularity more often than laypersons. In the Finnish texts, the prevalence of loaded language, guilt by association, Hitler card, ad hominem, *tu quoque*, poisoning the well, weak analogy, false dilemma/dichotomy, straw man and complex question were higher for laypersons than for politicians. In the Irish texts, appeal to personal incredulity occurred more often in political argumentation.

### Comparisons between countries

When the layperson opinions were compared between the countries, the Finnish texts contained higher prevalence of guilt by association, appeal to consequences, weak analogy and appeal to personal incredulity (Fig. 2). The same was observed for stereotyping/generalization and magical/religious thinking (Fig. 1). Appeal to popularity was more prevalent in the Irish texts. Regarding political debate, the Irish deputies showed higher prevalence of many types of arguments (loaded language, appeal to emotion, guilt by association, appeal to authority, appeal to popularity, ad hominem, *tu quoque,* appeal to ridicule, straw man, complex question) and of testimonials (Figs. 1 and 2).

### Comparisons between political parties

In the Finnish sample material, the prevalence of loaded language was the lowest and that of appeal to ridicule the highest in the contributions of Left Alliance deputies. Christian Democrats and Finns Party had the highest prevalence of testimonials and appeal to popularity. Features of magical/religious thinking were mainly documented in the contributions of Finns Party. Regarding Ireland, the prevalence of appeal to emotion, appeal to popularity, hasty conclusion/generalization, ad hominem, *tu quoque* and complex question were the highest in the contributions of United Left and independent deputies.

The data were also analyzed based on political spectrum (left–right axis). In Finland, the prevalence of loaded language, appeal to emotion and appeal to popularity were higher for the pooled center–right-wing parties, and the same was observed for the prevalence of appeal to authority in Ireland. The prevalence of testimonials, loaded language, appeal to emotion, appeal to popularity, ad hominem, *tu quoque*, hasty conclusion/generalization and complex question were higher for the pooled center–left-wing parties in Ireland.

## Discussion

### General findings

The abortion debate is often characterized by polarization [37] and misinformation [38], and the results of the present study confirmed these aspects regarding the Finnish and Irish abortion discourses. The right to life of the fetus and abortion as a human right of the woman were the two prominent perspectives found in the analysis (see also [39, 40]). The main finding was that the debates relied heavily on experiential thinking and emotional arguments instead of objective research data, confirming the first hypothesis. This was evident in the very high prevalence of testimonials, loaded language and appeals to emotion, regardless of the country or the author’s position. Not only did the laypersons but also the politicians justify their opinion based on narratives and other testimonials. The scientific knowledge about fetal brain function including the development of pain perception was scarcely mentioned, and a balanced discussion about the possible health risks of the abortion procedures was rare. There was often no genuine attempt to understand the position of the opponent, who was being caricatured and whose arguments both sides of the debate distorted. In many cases, the texts did not aim to resolve the deep disagreement but the debaters appeared to invest more effort into winning the dispute (see also [39]).

Testimonials were abundant in the abortion debate (see also [24, 25]) and appeared in the form of narratives, quotes and assertions without objective evidence, but presented as if they were indisputable facts. These included, for instance, personal experiences, stories of women who regretted or were empowered by their abortion, letters from traumatized midwives and referring to the tragic Irish cases associated with the restricted access to safe abortion. Smyth [37] has analyzed the “X case” in 1992 that is often cited in the Irish debate and represents an example of a narrative being used as a tool for argumentation. Individual case histories can be more effective “evidence” in persuasion of the audience than group statistics [19, 41], and they can make the issue personal (“If she was my daughter…”; [37]). While this was apparently a part of a successful strategy to amend legislation in 1992, it should be remembered that both sides of the debate utilize narratives that can reflect sincere experiences but would lead to opposite conclusions about abortion issues if they were used as a basis for decision-making. Thus, it would be very difficult to construct a coherent legislation based on them. Stories on “abortion survivors” provide examples of vivid pro-life narratives about people, whose mothers allegedly attempted to terminate their pregnancies but failed. A related strategy of promoting one’s case that is not based on research data is symbolic language in the form of metaphors, which can be useful in simplifying complicated issues, especially in the political arena [42].

Misconceptions about abortion are common and can derive from distorted (either deliberate or unintentional) interpretation of scientific literature and lack of source criticism [38]. Even though accurate information on induced abortion is widely available online, people are prone to confirmation bias and often seek information consistent with their existing beliefs while ignoring contradictory data [19]. This was evident when the studied texts discussed possible mental health consequences of abortion, such as the so-called post-abortion syndrome [38, 43]. Another strategy was to concentrate on surgical abortion, sometimes with graphic images, without acknowledging that the majority of abortions are performed medically, e.g., in Finland (96%; [16]) and England/Wales (55%; [44]). Similarly, pro-choice texts often emphasized that it would be inappropriate to cause guilt in the pregnant woman as the decision-making on abortion is always very difficult. In reality, not everyone struggles with the decision to choose a termination of pregnancy [45]. This feature of experiential thinking is accompanied with resistance to change, where data opposite to the debater's views fail to affect the opinion of the debater [19].

Many debaters utilized emotionally loaded language that can evoke negative feelings and shape the audience's attitudes on abortion [39, 46]. Appealing to emotions was very abundant in the studied debates (see also [24, 25] for examples in the UK and USA) and mostly presented as appeals to pity but appeals to anger and fear were also observed. Appealing to emotions is known to be common in political rhetoric [47]. The abortion issue was occasionally personalized, for instance, by asking if the opponent would force his wife or daughter to carry the fetus to term if the pregnancy was the result of rape. In many cases, debaters drew attention to the unfortunate circumstances of a person instead of the complex ethical situation of balancing the needs of the pregnant woman, the fetus and the society within the legislation. This strategy may solidify the popularity of an opinion among those who already agree but does not necessarily serve the cause of persuading others to change their position. In concert with the present results, the high frequency of emotional elements in abortion discourse was also previously emphasized [39]. Regarding other themes in public discourse, creationism represents another highly polarized topic, the debate of which relies on experiential thinking and emotional fallacies [20, 34]. Also in this case, the discussion has failed to convince the opposition to change their opinion suggesting that emotional argumentation does not advance the position of the debater among those who disagree with him/her.

### Comparison of political vs. layperson debates

It could be expected that politicians would be able to assess data that do not support one’s own worldview or preconceptions, as political decision-making should take into consideration the well-being of both sides. In accordance with the second hypothesis, the parliamentary discussions were evaluated to be of a higher quality compared to the layperson texts regarding the prevalence of experiential thinking and potential fallacies. No direct associations to Nazism were observed in the political speeches, but sometimes the opponent's opinion was connected to other unpopular phenomena, such as infanticide, gendercide, witch trials and past injustices committed against women in Ireland (Magdalene laundries, mother and baby homes, symphysiotomies), with guilt by association. Appeals to uncertain consequences were observed, e.g., regarding the passing of the Irish suicide clause. In this case, it was claimed that the clause would lead to normalizing suicide and to opening of floodgates to abortion on demand [48]. Here, the emotion resulting from the assertion of an unlikely consequence was used to persuade the opponent/audience.

While potentially fallacious argumentation was generally more prevalent in the layperson texts, there were some exceptions such as appeals to popularity in both countries. In Finland, deputies supporting CO referred to the majority of countries in Western Europe that had already introduced CO, without acknowledging the problems that emerged regarding the availability of abortion services [17]. In Ireland, opinion polls were used as a justification for or against the legislative reform. There has been discussion whether *ad populum* would be reasonable and not fallacious in the political debate [32, 49]. It is acknowledged that in democratic countries, policy makers are responsible for their voters and should, thus, pay attention to the public. On the other hand, opinion polls with simplified questions can be unreliable markers of real public sentiment over a complex issue, and the poll results can be interpreted based on personal preferences, i.e., with confirmation bias. In the study material, both sides of the debate occasionally interpreted similar poll results in a manner that the majority of the people would support the viewpoint of the debater. Although the differences in the prevalence of argumentative moves between the politicians and laypersons were more pronounced for Finnish contributions, it must be recalled that the shorter text lengths of the parliamentary transcripts could be a causative factor regarding this observation.

### Comparison of Finland and Ireland

The Irish abortion debate remains passionate [50], whereas the discussion in mainstream media is less active in Finland, possibly due to the long tradition of a liberal abortion legislation [5]. The present study observed that Irish debaters utilized appeals to popularity more often than those in Finland. One possible explanation could be in the association of the abortion discourse to nationalism. In the late twentieth century, the history of British oppression was tied to the abortion issue by associating England (the destination of abortion tourism) to brutal industrial capitalism and Ireland to the dying victim [37]. As an example, the English abortion policy was referred to as “the abortion mills of England [that] grind Irish babies into blood that cries out to heaven for vengeance” in one protest poster. The Finnish abortion debates analyzed also utilized appeals to popularity but were free from connections to national history or trauma. The earlier debate in Ireland on the “X-case” also included the concept of defending Christianity against barbarism [37]. In some cases, this type of rhetoric still continues, for instance, when past Christian values are described as “absolute” including the knowledge of abortion being wrong, and the loss of these values because of adherence to evolutionary theory (see also [34] for debate on creationism).

It is generally perceived that religiousness is lower in Finland with the Lutheran state church compared to Ireland, which has a nationalist popular church system where Catholicism has helped to preserve the cultural and national identity during the centuries of British rule [51, 52]. However, the referral to supernatural entities (mainly the Christian God and Jesus, i.e., traditional religious beliefs) as relevant for the argument was mostly present in Finnish blog texts. The past of the Irish abortion debate could shed some light on this finding. Smyth [37] noticed a change of arguments from the state being the defender of public morality to being the protector of the weak and vulnerable. In our opinion, this would fit the goals of both camps, as the pro-life fraction could be seen as defending the fetus as the abortion victim and the pro-choice camp as defending women’s rights. Thus, morality (religious doctrine) would be less openly discussed. Instead, appeal to people had apparently become a useful form of argument, and the same could still be observed in the present material in a refined form when using the appeals to polls as the voice of “the people”.

Another factor in the higher utilization of magical/religious thinking by Finnish layperson debaters could be the long tradition of Lutheran revivalist movements in Finland. The national Evangelical Lutheran Church includes several pietistic organizations that emphasize the Bible and personal commitment to the Christian religion [53]. While only a minority of Finnish people belong to a revivalist organization, many of those who are members still subscribe to the literal interpretation of the Bible including very conservative views on marriage, sexuality and abortion. In contrast, Catholicism has not produced similar revivalist organizations, although the Catholic Charismatic Renewal has gained popularity [54]. Still, it does not subscribe to as strict Biblical fundamentalism as, e.g., Pentecostalism, nor does the mainstream Catholic doctrine follow the literal interpretation of the Bible [55]. In this manner, while the general population in Finland is probably more secular than in Ireland, the revivalist writers contained in the sample material utilized Biblical references openly in their texts contributing to the observed difference between the countries.

Political debate is not independent of the attempts to influence it by the society. For instance, lobbying politicians is very professional in Ireland [56]. The present study did not focus on the lobbying or on its effects, but it is feasible to assume that some layperson texts or similar ones could have been used as material for lobbying, as their obvious function was to affect the opinions of the readers, some of which may well have been politicians. If these texts were used for such purposes, their success might not necessarily be very good, as the present analysis reveals, how the debate recycles similar emotional arguments and fallacies, which can derive from experiential thinking patterns [20] and mostly enforce the opinions of those who already agree on the issue. However, the presence of lobby groups shows that the nature of the abortion debate is also multifaceted in Ireland and much more complex than it would be in a strictly “Catholic and conservative” country.

The effects of political orientation on argumentative strategies and on the features of experiential thinking were not easy to interpret and they also differed between the countries. Some differences in the choice of arguments can be due to a few individual deputies making several emotional addresses and, thus, dominating the parliamentary speeches of their own party. Deputies of Christian Democrats and Finns Party utilized testimonials and appeals to popularity most often, whereas in Ireland, the prevalence of many argumentative moves were higher in the speech acts of deputies of United Left. It must be emphasized that the sample sizes were relatively small in these comparisons and, for this reason, these results are regarded as secondary findings at the moment. While the preliminary results on different argumentative strategies between parties are intriguing, a more thorough analysis of them requires further research.

### Potentially fallacious argumentation

It is not always straightforward to discern fallacious arguments from reasonable ones. This difficulty was also present during the present research process as the analyzed texts were sometimes ambiguous and this type of analysis is always context-dependent and somewhat subjective. It can still be asserted that instead of defending one’s position with unbiased research data or evaluations of both sides of the controversy, there were very many instances of argumentation based on experiential thinking patterns that could be classified fallacious. For example, the use of testimonials often leads to appealing to authorities [20] and, in the present study, these included appeals to anonymous medical experts, the Bible and citing religious leaders. There were also abundant references to legitimate authorities regarding abortion either as a medical procedure or as a legislative issue, especially in the discussion in the Dáil Éireann. It was also observed how the texts utilized testimonials and narratives but neglected data that did not support the debater's position (confirmation bias). While these strategies of discourse can be persuasive, in these cases the authors employed experiential instead of rational thinking.

Assessing the fallacies and thinking patterns leads to the question, if the debate on induced abortion is at all useful when trying to convince those of the opposing viewpoint. Based on the present analysis, it seems that, despite the lively discussion, the arguments used in the abortion controversy hardly evolve, but similar claims and emotional strategies occur in diverse texts and parliamentary speeches. It seems that the situation has not changed very much, as the Irish debate has remained polarized for decades, with strong indications of “us vs. them” [37]. The argumentative tools used by both laypersons and politicians do not seem to attain the goal of increasing the number of proponents on the debater's side. If resolving the disagreement or persuading the opposition to one’s viewpoint were a goal of the debate, the current arguments by the proponents of either liberal or restrictive abortion legislation fail miserably.

## Conclusions

The studied abortion debates relied heavily on experiential thinking and emotional arguments instead of objective research data in both countries. This was evident in the very high prevalence of testimonials, narratives, loaded language and appeals to emotion in both political and layperson debates regardless of the debater's position on abortion issue. The abortion debate mostly reinforces the opinions of one's peer group rather than convinces the opposite party to change their position. The stalemate and continuation of the same arguments being repeated could be associated with experiential thinking and emotional argumentative strategies in both political and layperson debates. If the debaters familiarized themselves with these concepts, it might be possible to reach a common ground more easily instead of enforcing the differences.

## Abbreviations

CO: Conscientious objection

## References

[1] Kaposy C (2012). Two stalemates in the philosophical debate about abortion and why they cannot be resolved using analogical arguments. Bioethics.

[2] Stephens M, Jordens CFC, Kerridge IH, Ankeny RA (2010). Religious perspectives on abortion and a secular response. J Relig Health.

[3] Citizens Information Board (2015). Entitlement to health services.

[4] Ministry of Social Affairs and Health (2017). Social and health services.

[5] Ministry of Justice (1970). Law no 239 of 24 March 1970 on the interruption of pregnancy.

[6] Electronic Irish Statute Book (2013). Protection of life during pregnancy act 2013.

[7] Bloomer F, O’Dowd K (2014). Restricted access to abortion in the Republic of Ireland and Northern Ireland: exploring abortion tourism and barriers to legal reform. Cult Health Sex.

[8] Government of Ireland (2017). Census 2016 summary results – part 1.

[9] Atlas of European Values. 2011. http://www.atlasofeuropeanvalues.eu/new/zieeuropa.php?year=2008. Accessed 6 June 2016.

[10] Statistics Finland (2017). Population.

[11] Taylor M (2015). Women's right to health and Ireland's abortion laws. Int J Gynecol Obstet.

[12] Aiken ARA, Gomperts R, Trussell J (2016). Experiences and characteristics of women seeking and completing at-home medical termination of pregnancy through online telemedicine in Ireland and Northern Ireland: a population-based analysis. BJOG.

[13] Nieminen P, Lappalainen S, Ristimäki P, Myllykangas M, Mustonen A-M (2015). Opinions on conscientious objection to induced abortion among Finnish medical and nursing students and professionals. BMC Med Ethics.

[14] Tiitinen A. Induced termination of pregnancy. Duodecim Terveyskirjasto. 2016. http://www.terveyskirjasto.fi/terveysportti/tk.koti?p_artikkeli=dlk00166. Accessed 16 May 2017. [in Finnish].

[15] Parliament of Sweden (1974). The abortion act (1974:595): Swedish code of statutes 1974:595.

[16] Heino A, Gissler M (2016). Induced abortions 2015.

[17] Heino A, Gissler M, Apter D, Fiala C (2013). Conscientious objection and induced abortion in Europe. Eur J Contracept Reprod Health Care.

[18] Epstein S (1994). Integration of the cognitive and the psychodynamic unconscious. Am Psychol.

[19] Lindeman M (1998). Motivation, cognition and pseudoscience. Scand J Psychol.

[20] Nieminen P, Ryökäs E, Mustonen A-M (2015). Experiential thinking in creationism—a textual analysis. PLoS One.

[21] Fogelin RJ, Duggan TJ (1987). Fallacies. Argumentation.

[22] van Eemeren FH, Grootendorst R (1992). Argumentation, communication, and fallacies: a pragma-dialectical perspective.

[23] Yap A (2013). Ad hominem fallacies, bias, and testimony. Argumentation.

[24] Schonhardt-Bailey C (2008). The congressional debate on partial-birth abortion: constitutional gravitas and moral passion. Br J Political Sci.

[25] Childs S, Evans E, Webb P. “Quicker than a consultation at the hairdressers”: abortion and the Human Fertilisation and Embryology Act 2008. New Genet Soc. 2013;32:119–34.

[26] Petchesky RP (1987). Fetal images: the power of visual culture in the politics of reproduction. Fem Stud.

[27] McNeil M, Franklin S, Lury C, Stacey J (1991). Putting the Alton Bill in context. Off-Centre: feminism and cultural studies.

[28] Michaels MW, Morgan LM, Michaels MW (1999). Fetal galaxies: some questions about what we see. Fetal subjects, feminist positions.

[29] Boucher J (2004). Ultrasound: a window to the womb?: Obstetric ultrasound and the abortion rights debate. J Med Humanit.

[30] Walton DN (1996). Argumentation schemes for presumptive reasoning.

[31] Walton D (1999). Historical origins of *argumentum ad consequentiam*. Argumentation.

[32] Walton D, Fontana B, Nederman CJ, Remer G (2004). Criteria of rationality for evaluating democratic public rhetoric. Talking democracy: historical perspectives on rhetoric and democracy.

[33] Sahlane A (2012). Argumentation and fallacy in the justification of the 2003 war on Iraq. Argumentation.

[34] Nieminen P, Mustonen A-M (2014). Argumentation and fallacies in creationist writings against evolutionary theory. Evolution: Education and Outreach.

[35] Bennett B (2015). Logically fallacious: the ultimate collection of over 300 logical fallacies.

[36] Fallacies DB (2016). The internet encyclopedia of philosophy.

[37] Smyth L (1998). Narratives of Irishness and the problem of abortion: the X case 1992. Fem Rev.

[38] Rowlands S (2011). Misinformation on abortion. Eur J Contracept Reprod Health Care.

[39] Mazilu S. Strategic use of emotional terms in ethical argumentation on abortion. L’Analisi Linguistica e Letteraria. 2008;XVI:683–95.

[40] Larsson S, Eliasson M, Klingberg Allvin M, Faxelid E, Atuyambe L, Fritzell S (2015). The discourses on induced abortion in Ugandan daily newspapers: a discourse analysis. Reprod Health.

[41] Dahlstrom MF (2014). Using narratives and storytelling to communicate science with nonexpert audiences. PNAS.

[42] Mio JS (1997). Metaphor and politics. Metaphor Symb.

[43] van Ditzhuijzen J, ten Have M, de Graaf R, Lugtig P, van Nijnatten CHCJ, Vollebergh WAM (2017). Incidence and recurrence of common mental disorders after abortion: results from a prospective cohort study. J Psychiatr Res.

[44] Department of Health (2016). Abortion statistics, England and Wales: 2015.

[45] Ralph LJ, Greene Foster D, Kimport K, Turok D, Roberts SCM (2017). Measuring decisional certainty among women seeking abortion. Contraception.

[46] Mikołajczak M, Bilewicz M (2015). Foetus or child? Abortion discourse and attributions of humanness. Br J Soc Psychol.

[47] Jerit J (2004). Survival of the fittest: rhetoric during the course of an election campaign. Polit Psychol.

[48] de Londras F, Graham L (2013). Impossible floodgates and unworkable analogies in the Irish abortion debate. Irish J Legal Studies.

[49] Andone C (2016). Argumentative patterns in the political domain: the case of European parliamentary committees of inquiry. Argumentation.

[50] Qadir Z (2013). Ireland’s abortion debate. Lancet.

[51] Höllinger F, Haller M, Valle-Höllinger A (2007). Christian religion, society and the state in the modern world. Innovat Eur J Soc Sci Res.

[52] Ketola K, Ketola K, Niemelä K, Palmu H, Salomäki H (2011). Religiosity of Finnish people. Religion in the life of Finnish people: religious upbringing, morality, happiness and tolerance with international comparison. Yhteiskuntatieteellisen tietoarkiston julkaisuja 9.

[53] Talonen J, Hytönen M (2004). Interpretation of the Bible in Finnish revivalist movements. Bible and faith of the church today.

[54] Daniel K (2010). An assessment of the Catholic charismatic renewal towards peaceful co-existence in the Roman Catholic Church. Int J Sociol Anthropol.

[55] Benedict XVI (2010). Post-synodal apostolic exhortation *verbum domini* of the holy father Benedict XVI to the bishops, clergy, consecrated persons and the lay faithful on the Word of God in the life and mission of the church.

[56] Murphy G, Bitonti A, Harris P (2017). Ireland. Lobbying in Europe: public affairs and the lobbying industry in 28 EU countries.

[57] Creighton L (2013). Dáil Éireann debate.

[58] Pinto S (2015). Six outrageous facts about abortion in Ireland.

[59] Ebeling M (2013). Amnesty swallowed a camel – but strained at a gnat!.

[60] Daly C (2015). Dáil Éireann debate.

[61] Cromie C (2015). Abortion and “healthcare”: dispelling the myths.

[62] Räsänen P (2015). Parliament of Finland debate.

[63] O’Sullivan M (2013). Dáil Éireann debate.

[64] Soini T (2008). Parliament of Finland debate.

[65] O’Gorman T (2013). Architect of Britain’s abortion law horrified at sex selective abortions.

[66] Soini T (2009). Parliament of Finland debate.

[67] Lehkamo A (2011). Abortion – no thank you.

[68] Paavola P (2012). Abortion.

[69] Soini T (2015). Parliament of Finland debate.

[70] Daly C (2014). Dáil Éireann debate.

[71] Coppinger R (2015). Dáil Éireann debate.

[72] Daly C (2014). Dáil Éireann debate.

[73] Ui Bhriain N (2015). Attempts to write the baby out of the abortion debate won’t succeed.

[74] Varadkar L (2015). Dáil Éireann debate.

[75] Maloney E (2013). Dáil Éireann debate.

[76] Anonymous (2010). Abortion equals murder, full stop.

[77] Tolppanen M (2015). Parliament of Finland debate.

[78] Niikko M (2015). Parliament of Finland debate.

[79] Forrestal M (2015). How Amnesty betrayed its members to push abortion.

[80] Quinn D (2013). Incoherence and illogic in the Dail abortion debate.

[81] Salonen K (2015). Parliament of Finland debate.

[82] Kari E (2015). Why is only the life of an unborn child holy to the men of Finns party?.

